# The complete chloroplast genome sequence of *Stilpnolepis centiflora* (Asteraceae), an endemic desert species in Northern China

**DOI:** 10.1080/23802359.2020.1829127

**Published:** 2020-10-09

**Authors:** Xiaojun Shi, Kaiqing Xie

**Affiliations:** Xinjiang Key Laboratory of Grassland Resources and Ecology and Ministry of Education Key Laboratory for Western Arid Region Grassland Resources and Ecology, College of Grassland and Environment Sciences, Xinjiang Agricultural University, Urümqi, People’s Republic of China

**Keywords:** *Stilpnolepis centiflora*, chloroplast genome, Illumina sequencing, phylogenetic analysis

## Abstract

*Stilpnolepis centiflora*, a monotypic genus of the Asteraceae, is an endemic desert species in Northern China. However, information on the chloroplast (cp) genome of this species is limited. In this study, we present the complete chloroplast genome sequence of *S. centiflora* obtained by high-throughput nextgeneration sequencing technology. The whole cp genome was 151017 bp long and comprised 133 genes, including 88 proteincoding genes, 37 tRNA genes, and 8 rRNA genes. The *S. centiflora* cp genome had a GC content of 37.35%. Phylogenetic tree revealed that *S. centiflora* was closely related to the taxa in the genus *Artemisia* and *Chrysanthemum*. Our results would be helpful for species identification and promote our understanding of the phylogeny of *Stilpnolepis* within the Asteraceae family.

*Stilpnolepis centiflora*, a monotypic genus of the Asteraceae, is an endemic desert species in Northern China (Zhao 1996, 1997). It is a small annual herb which is endemic and disjunction distribution species between five deserts (Kubuqi, Mu Su, Badain Jaran, Ulan Buh and Tengger) in Northern China, which especially occurs around on mobile sand dunes and flat sand sheets between dunes (Zhao 1996). It has been proved to have potential medicinal value because some extracted substances from it have potent antiviral, expectorant and antiasthmatic (Duan et al. 1996). Previous studies have been conducted on phylogenetic relationships between *S. centiflora* and other related species by using ribosomal DNA data (ITS) (Watson et al. 2002). However, there was no report about the chloroplast genome data of *S. centiflora*. In this study, we presented the first complete chloroplast genome sequence of *S. centiflora* based on the Illumina paired-end sequencing data. Furthermore, we assessed phylogenetic relationships between *S. centiflora* and other related species, which may help us to have a comprehensive knowledge on the phylogeny of *S. centiflora*.

Fresh leaves of *S. centiflora* were obtained from the Shapotou, Zhongwei, Ningxia Province of China (104.93°E, 37.45°N) and dried immediately by silica gels. The voucher specimen also deposited at the Xinjiang Agricultural University Herbarium (XN2020072001). Total genomic DNA of *S. centiflora* was isolated from leaf tissues using the modified CTAB method (Doyle and Doyle 1987). The shotgun library with insert size of 370 bp fragments was constructed and genome sequencing was performed using the Illumina HiSeq Platform (Illumina, San Diego, CA) at Genepioneer Biotechnologies Inc., Nanjing, China. Approximately 5.0 GB of clean data were yielded. The programs SPAdes (Bankevich et al. 2012) and CpGAVAS (Liu et al. 2012) were used to assemble and annotate, respectfully. The cp genome of *Artemisia selengensis* (MH042532.1) (Peng et al. 2018) was included as the initial reference. The annotated genomic sequence has been submitted to GenBank (accession number MT830619).

The complete chloroplast genome of *S. centiflora* which contained a typical conserved quadripartite structure, with a LSC region of 82,782 bp, a SSC region of 18,395 bp, and a pair of IRs regions of 24,920 bp, was 1,51,017 bp in length. The overall GC content was 37.35%, whereas the GC content in the LSC, SSC, and IR regions were 35.41, 30.51, and 43.09%, respectively. A total of 133 genes were identified, including 88 proteincoding, 37 transfer RNA and eight ribosome RNA genes. Among these genes, fifteen genes (*atpF*, *ndhA*, *ndhB*, *petB*, *petD*, *rpl16*, *rpl2*, *rpoC1*, *rps16*, *trnA-UGC*, *trnG-UCC*, *trnI-GAU*, *trnK-UUU*, *trnL-UAA*, *trnV-UAC*) contained a single intron and three genes (*rps12*, *ycf3* and *clpP*) contained two introns.

The aligned complete chloroplast genome sequences of *S. centiflora* and 26 other species belonging to the Asteraceae family by MAFFT (Katoh and Standley 2013) were used for phylogenetic analysis, and *Mikania micrantha* and *Guizotia abyssinica* of Asteraceae family were used as out-group. The phylogenetic tree was constructed using maximum-likelihood implemented in IQ-TREE 1.6.2 (Nguyen et al., 2015) under the TVM + F + R2 nucleotide substitution model, which was selected by ModelFinder (Kalyaanamoorthy et al. 2017). Support for the inferred ML tree was inferred by bootstrapping with 1000 replicates. Phylogenetic analysis showed that *S. centiflora* was closely related to the taxa in the genus *Artemisia* and *Chrysanthemum* (Figure 1). Our data are helpful for species identification and promote our understanding of the phylogeny of *Stilpnolepis* within the Asteraceae family.

**Figure 1.**
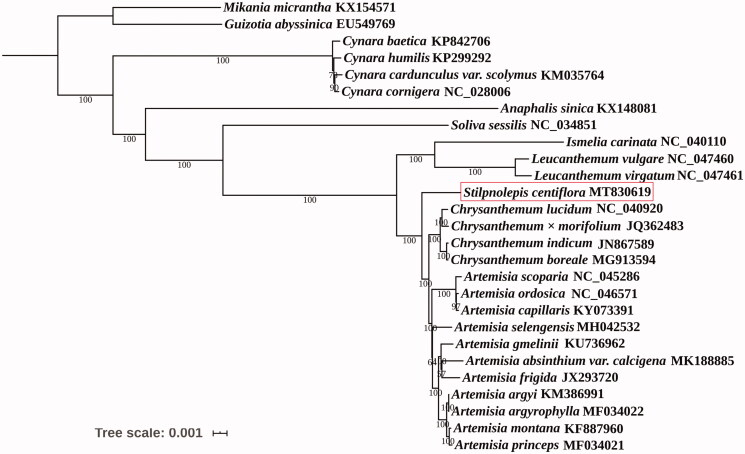
Maximum-likelihood phylogenetic tree based on the complete chloroplast genome sequences of *Stilpnolepis centiflora* and 26 other species. Values along branches correspond to ML bootstrap percentages.

## Data Availability

The data that support the findings of this study are openly available in GenBank of NCBI at https://www.ncbi.nlm.nih.gov, reference number MT830619.
